# Reirradiation for diffuse intrinsic pontine glioma in an upper middle-income country: does full dose improve outcomes?

**DOI:** 10.1007/s00381-026-07390-x

**Published:** 2026-08-20

**Authors:** Natália Dassi, Maria Luisa Sucharski Figueiredo, Francine Tesser Gamba, Marcos Devanir Silva da Costa, Ana Carolina dos Santos Torquato, Jessica Benigno Rodrigues, Ronaldo Modesto de Souza Filho, Sergio Cavalheiro, Patricia Alessandra Dastoli, Frederico Adolfo Silva, Carlota Vitoria Blassioli Moraes, Silvia Regina Caminada de Toledo, Nasjla Saba Da Silva, Michael Jenwei Chen, Andrea Maria Cappellano

**Affiliations:** 1 Department of Pediatric Oncology, IOP-GRAACC-Federal University of Sao Paulo, Pedro de Toledo Street, 572, Sao Paulo, 04039001 Brazil; 2 Department of Radiation Oncology, IOP-GRAACC-Federal University of Sao Paulo, Sao Paulo, Brazil; 3 Genetics Laboratory, IOP-GRAACC-Federal University of Sao Paulo, Sao Paulo, Brazil; 4 National Science and Technology Institute for Children’s Cancer Biology and Pediatric Oncology – INCT BioOncoPed, Sao Paulo, Brazil; 5 Department of Neurosurgical Oncology, IOP-GRAACC-Federal University of Sao Paulo, Sao Paulo, Brazil; 6 Department of Pathology , IOP-GRAACC-Federal University of Sao Paulo, Sao Paulo, Brazil; 7 Department of Oncologic Radiology, IOP-GRAACC-Federal University of Sao Paulo, Sao Paulo, Brazil

**Keywords:** Diffuse intrinsic pontine glioma, Tumor biopsy, Molecular alterations

## Abstract

**Purpose:**

Reirradiation (reRT) during diffuse intrinsic pontine glioma (DIPG) progression has emerged as a potential treatment strategy for prolonging survival.

**Methods:**

This retrospective, single-center observational study was conducted on children diagnosed with DIPG between 2010 and 2025, and data from 58 were collected. Doses for reRT were as follows: 50–54 Gy (1.8–2.0 Gy daily/fractions) or 39 Gy (3 Gy daily/fractions). Overall survival (OS) was defined as the time from diagnosis to the date of death and reRT OS, reirradiation starting to death. The Kaplan–Meier method and log-rank test were used for survival analysis.

**Results:**

The median age was 7.1 years (range 2.9–16.2 years). Twenty-six patients underwent a biopsy. The *H3K27M* mutation was the most prevalent in our study, followed by *TP53* altered gene. Twenty-two patients underwent reRT at progression. The median time from initial treatment to reRT was 9.9 months (range 6.7 to 20.5 months). Seven of the 22 patients received reRT at a dose of 39 Gy while 14 at doses of 50–54 Gy. The median OS from reRT to death was 5.8 months (range, 0.9–18.3 months). Patients who received reRT had a longer OS compared to those who did not (median OS of 17.9 versus 9.8 months; *p* = 0.005). Longer time for progression (> 9 months) and dose of reRT > 50 Gy were statistically significantly correlated with better OS. Reirradiation was generally well-tolerated by all patients.

**Conclusions:**

This study offers molecular insights into this tumor type and presents a therapeutic option with higher nominal doses reRT within a palliative context to prolong survival of these patients.

## Introduction

Diffuse intrinsic pontine gliomas (DIPGs) are aggressive pediatric brainstem tumors, constituting approximately 80% of all brainstem gliomas [1]. They are now classified as diffuse midline gliomas in the World Health Organization classification of central nervous system tumors, specifically designated as diffuse midline glioma, *H3 K27-altered* [2]. This reclassification reflects the identification of a unifying molecular hallmark — H3 K27 alteration — that defines this entity across midline structures, even as additional biological diversity within this group continues to be characterized [2–4].

The diagnosis of DIPG is primarily clinical, based on a classic triad of symptoms: cranial nerve palsy (most commonly involving cranial nerve VI), long tract signs (pyramidal tract dysfunction), and cerebellar ataxia. Symptoms typically last less than 3 months. Magnetic resonance imaging (MRI) findings typically reveal a diffusely infiltrating tumor involving over 50% of the pons. This tumor is characterized by hyperintensity on T2-weighted and FLAIR sequences and hypointensity on T1-weighted images. Although traditionally diagnosed without histological confirmation due to the eloquence of the brainstem, advances in the safety of neurosurgical biopsy have enabled tissue sampling, significantly contributing to our understanding of the underlying molecular biology of these tumors [1, 5–8].

Conventionally, fractionated radiotherapy at a dose of 54–60 Gy is the standard treatment, with median progression-free and overall survival (OS) ranging from 6 to 12 months [9, 10]. Reirradiation (reRT) during DIPG progression has emerged in recent years as a potential treatment strategy for prolonging survival [11–13]. Doses for reRT usually range from 18 to 36 Gy, with no literature reporting doses of 50 Gy or higher. Moreover, the dose–response relationship is not well established [9].

The main aim of this study was to evaluate the role of reirradiation post-disease progression on the survival of patients treated at a single center. A secondary aim was to examine the clinical and molecular features, treatment-induced toxicities, and neurological manifestations of patients with DIPG who underwent either a single round of radiotherapy or received a second course of irradiation.

## Methods

This retrospective, single-center observational study was conducted on children diagnosed with DIPG between 2010 and 2025, with data analyzed in May 2025. The study was conducted at the Pediatric Oncology Institute (IOP-GRAACC), affiliated with the Federal University of São Paulo. Clinical information was extracted from patients’ medical records. The study design received approval from the Ethics Committee, and informed consent was obtained from patients who were still alive at the end of the follow-up period.

### Diagnosis

The diagnosis of DIPG was confirmed based on the clinical and radiographic features and/or biopsy findings. The initial staging included craniospinal MRI scans with and without contrast administration. Disease progression was defined as clinical or neurological deterioration confirmed by MRI, manifested by an increase in tumor volume and/or T2/FLAIR changes in non-enhancing tumors. All initial, postoperative, and progressive MRI scans were evaluated by a single neuroradiologist at our institution.

### Tumor biopsy

Stereotactic biopsies were performed via posterior fossa approach using a frameless neuronavigation system (Brainlab® Varioguide system). The head was immobilized using a DORO® system and positioned prone with slight flexion. Entry points were marked below the transverse sinus and lateral to the sigmoid sinus. The needle trajectory was planned through the cerebellar hemisphere, targeting the same side where the patient exhibited motor or cranial nerve deficits. Necrotic areas on imaging were avoided to minimize bleeding risks during the procedure. A minimum of 6–8 tissue samples were obtained to ensure an adequate amount of material for molecular analyses, including next-generation sequencing, in addition to standard histopathological evaluation.

### Pathology and molecular analysis

All histopathological diagnoses underwent review by a pathologist at our institution. Immunohistochemistry (IHC) was used to analyze *H3 K27M* and *TP53* protein expressions. Freshly frozen tumor samples were analyzed through targeted sequencing using the OCCRA (Oncomine™ Childhood Cancer Research Assay–Chef-ready - (Thermo Fisher Scientific) panel, encompassing 255 tumor-associated genes. The freshly frozen tumor samples utilized in this study were sourced from the Biobank of the Pediatric Oncology Institute GRAACC/UNIFESP (National Commission of Ethics in Research, CONEP B-053).

### Radiotherapy

The RT modality utilized for all treated patients was VMAT. The initial course of irradiation (RT1) consisted of a dose of 54 Gy delivered in 1.8 Gy daily fractions. For the reirradiation course (RT2), two schemes were employed: (1) 50–54 Gy in 1.8–2.0 Gy daily fractions or (2) 39 Gy in 3 Gy daily fractions. While a complete reRT dose of 50–54 Gy was preferred, the 39 Gy option was administered based on the child’s performance, as determined by the radiation oncologist, and the necessity of anesthesia. A minimum interval of 6 months following the first RT course was mandated before initiating RT2. The concurrent use of irradiation and systemic agents was permissible during any of the irradiation courses, based on the clinician’s experience. None of these agents constitutes established standard of care, and their use reflects the heterogeneous and evolving therapeutic landscape over the 15-year study period. In cases of disease progression, the clinical target volume for RT2 encompassed solely the tumor, delineated by FLAIR or T2-weighted MR images. Daily image guidance with cone-beam CT was mandatory for RT2. Adverse events were documented according to the Common Terminology Criteria for Adverse Events, reported retrospectively, using version 5.0.

### Survival analysis

Event-free survival (EFS) was defined as the time from diagnosis to the date of death or progression following RT1. Overall survival was defined as the time from diagnosis to the date of death, while reRT OS was measured from reirradiation starting to death. The Kaplan–Meier method and log-rank test were used for survival analysis.

## Results

Fifty-eight patients were included, with 32 being male. The median age was 7.1 years (range 2.9–16.2 years). Vomiting was the most common symptom at diagnosis (30/58, 51.7%), followed by cranial nerve alterations (23/58, 39.6%). The patient characteristics are summarized in Table 1.

**Table 1:** Patient characteristics

	All patients (*n* = 58)
Gender	
Female	26 (44.8%)
Male	32 (55.2%)
Age	
Median	7.1 years
Range	2.9–16.2 years
Biopsy	26/58 (44.8%)
H3K27M-mutated	24/26 (92.3%)
TP53 altered	7/26 (26.9%)
RT1	
54 Gy	58/58 (100%)
Additional treatments	39/58 (67.2%)
RT1 + RT2	22/58 (37.9%)
39 Gy*	8/22 (36.4%)
50 Gy	10/22 (45.4%)
54 Gy	4/22 (18.2%)
Additional treatments	17/22 (77.3%)
Time for progression	
Median	9.5 months
Range	3-35.2 months

*One patient did not complete RT2 due to clinical deterioration

Twenty-six patients underwent a biopsy: 25 at diagnosis and one after progression. Three patients experienced exacerbated facial paralysis post-biopsy; however, recovery ensued within 72 h following the procedure. The *H3K27M* mutation was the most prevalent in our study, identified in 24 out of 26 (92.3%) biopsy cases, as confirmed by IHC and NGS results. Among the 22 samples analyzed by NGS, the *H3.3* mutation was detected in twelve patients, *H3C2* and *H3.1* in four and one case each. TP53 ranked as the second most frequently altered gene, observed in seven cases ( 7/26 (26.9%) . Additional NGS findings comprised mutations in *ACVR1* and *PIK3CA*, amplifications of *KIT* and *PDGRFA*, and a *BRAFV600* mutation in a single case.

**Fig. 1:**
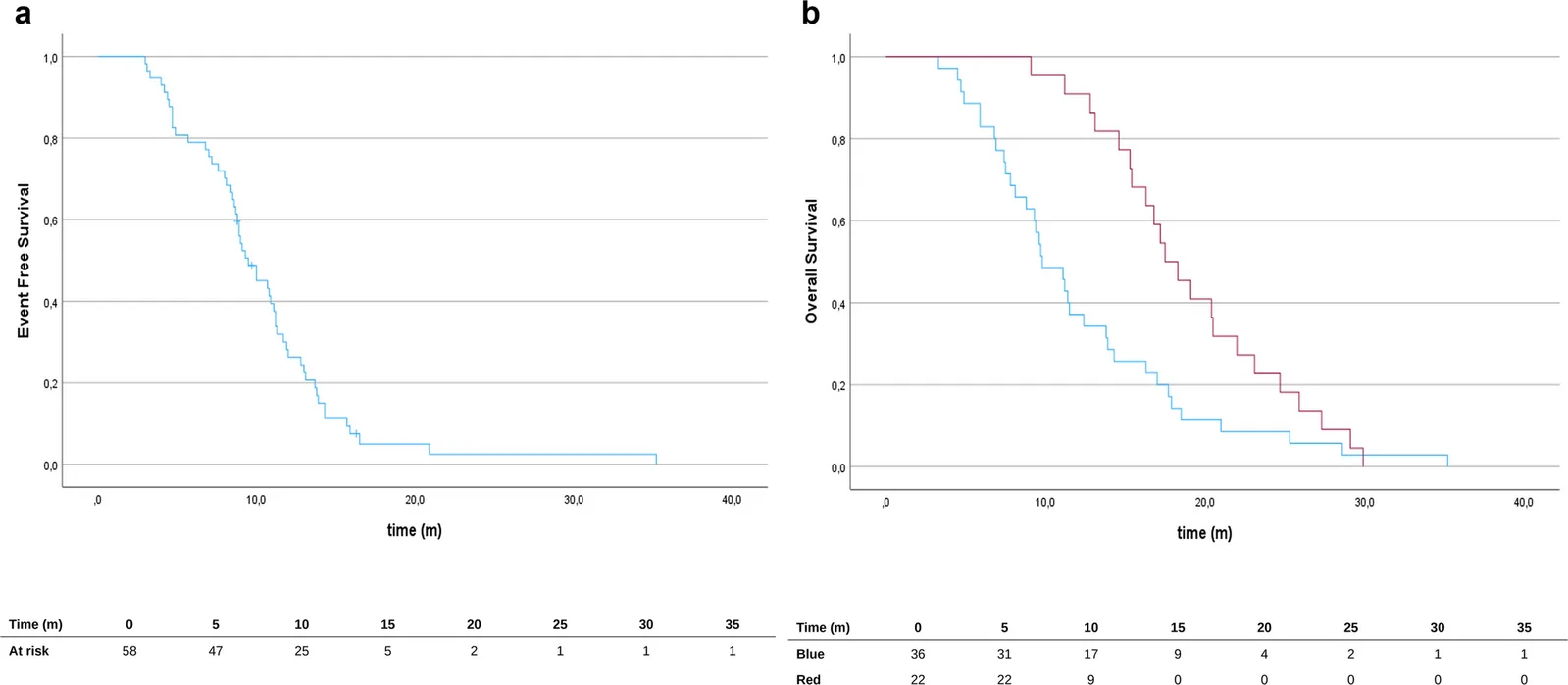
Event free survival after RT1 (**a**) and overall survival after RT1 and RT1 + RT2 (**b**). **a** Event free survival after RT1 (*n* = 58). **b** Overall survival RT1 (*n* = 36) and RT1 + RT2 (*n* = 22). Red line, RT1 + RT2; blue line, RT1 only

The median event-free survival (EFS) after RT1 (*n* = 58) was 9.5 months (range 3–35.2 months) (Fig. 1a). Thirty-six patients underwent RT1 exclusively. Among them, 30 (83.3%) were administered valproic acid as a histone inhibitor, five (13.8%) were given bevacizumab, five (13.8%) were treated with nimotuzumab, and one (2.8%) received nivolumab and ipilimumab.

Twenty-two patients underwent RT1 + RT2 (Table 2). For reRT, all patients showed progressive neurological symptoms, and disease progression was confirmed by MR features. The median time from initial treatment to reirradiation was 9.9 months, ranging from 6.7 to 20.5 months. Seven of the 22 patients received RT2 at a dose of 39 Gy (13 fractions of 300 cGy), while 14 received RT2 at doses of at least 50 Gy. One patient stopped the reRT with 26 Gy due to deteriorating clinical status. Five of the 22 (22.7%) patients received reirradiation alone, 8/22 (36.4%) received valproic acid, 8/22 (36.4%) bevacizumab, 1/22 (4.5%) nivolumab, and 1/22 (4.5%) received ONC201.

**Table 2:** Characteristics of patients submitted to reirradiation

Age at diagnosis (years)	Months from diagnosis to relapse	Time from RT1 to RT1 + RT2 (months)	Performance Status pre-RT2	RT2 dose (Gy)	Additional treatment at RT2	Symptoms improvements	Adverse effects	Months from RT1 + RT2 to death	Follow-up (months)	Biopsy	NGS
3.8	10.3	8.4	< 50	39	No	Yes	No	3.6	15.3	Yes	ACVR1; H3C2; PIK3CA
8.9	11.7	11.2	> 50	50	VPA	Yes	No	18.3	29.1	No	-
5	15.7	11.8	> 50	54	No	Yes	No	4.8	25.9	Yes	H3.3
3.7	7.6	6.7	> 50	54	VPA	No	No	4.6	13.1	No	-
3.6	13.8	12.6	> 50	54	VPA	Yes	No	5.9	20.5	No	-
10.6	8.4	8.9	> 50	54	No	No	No	7.6	16.8	No	-
9.7	9.1	8.6	< 50	39	No	Yes	No	0.9	11.2	No	-
3.7	11.1	9.9	< 50	26*	No	No	No	3.6	15.4	Yes	H3.3
6.9	13	11	> 50	50	No	Yes	No	10.8	24.7	Yes	H3.1
6.7	20.8	20.5	< 50	39	VPA	Yes	No	10.3	22	Yes	H3C2, TP53
9	9	8.3	> 50	50	VPA	Yes	No	7.3	17.2	No	-
3.4	14.3	13.1	< 50	39	VPA	Yes	No	3.3	18.3	No	-
10.1	12.8	12.4	> 50	50	Bevacizumab	Yes	No	10	23.1	No	-
3.7	10.7	9.9	> 50	50	Bevacizumab	Yes	No	7	19.1	Yes	H3.1
6	7.1	6.9	> 50	50	Bevacizumab	Yes	No	8.7	17.5	No	-
4.5	8.5	8.2	> 50	50	Bevacizumab	Yes	No	3.4	12.8	Yes	KIT/PDGFRA amplification; TP53
8.2	8.8	7.7	< 50	39	VPA + bevacizumab	No	No	5.6	14.6	No	-
3.6	15.8	14.2	< 50	39	VPA + bevacizumab	No	No	4.3	20.4	Yes	H3.3
8	8	7.2	> 50	50	Bevacizumab	Yes	No	18.3	27.3	Yes	H3.3
13.8	9.7	7	< 50	39*	No	No	No	2	10.4	Yes	H3.3; TP53
5.9	7.8	14	> 50	50	No	Yes	No	2.9	16.6	Yes	ACVR1; H3C2
5	12	11.7	> 50	50	Bevacizumab	Yes	No	17.8	29.9	Yes	PIK3CA; ASXL1; TP53

*One patient stopped the reRT with 26 Gy due to deteriorating clinical status. *VPA* valproic acid

The median OS from RT2 to death was 5.8 months (range, 0.9–18.3 months). Patients who received RT2 (*n* = 22) had a longer OS compared to those who did not receive RT2 (RT1 only) (*n* = 36), with a median OS of 17.9 months versus 9.8 months (*p* = 0.005) (Fig. 1b), even when no distinction was made between the different RT2 dose schedules.

**Fig. 2:**
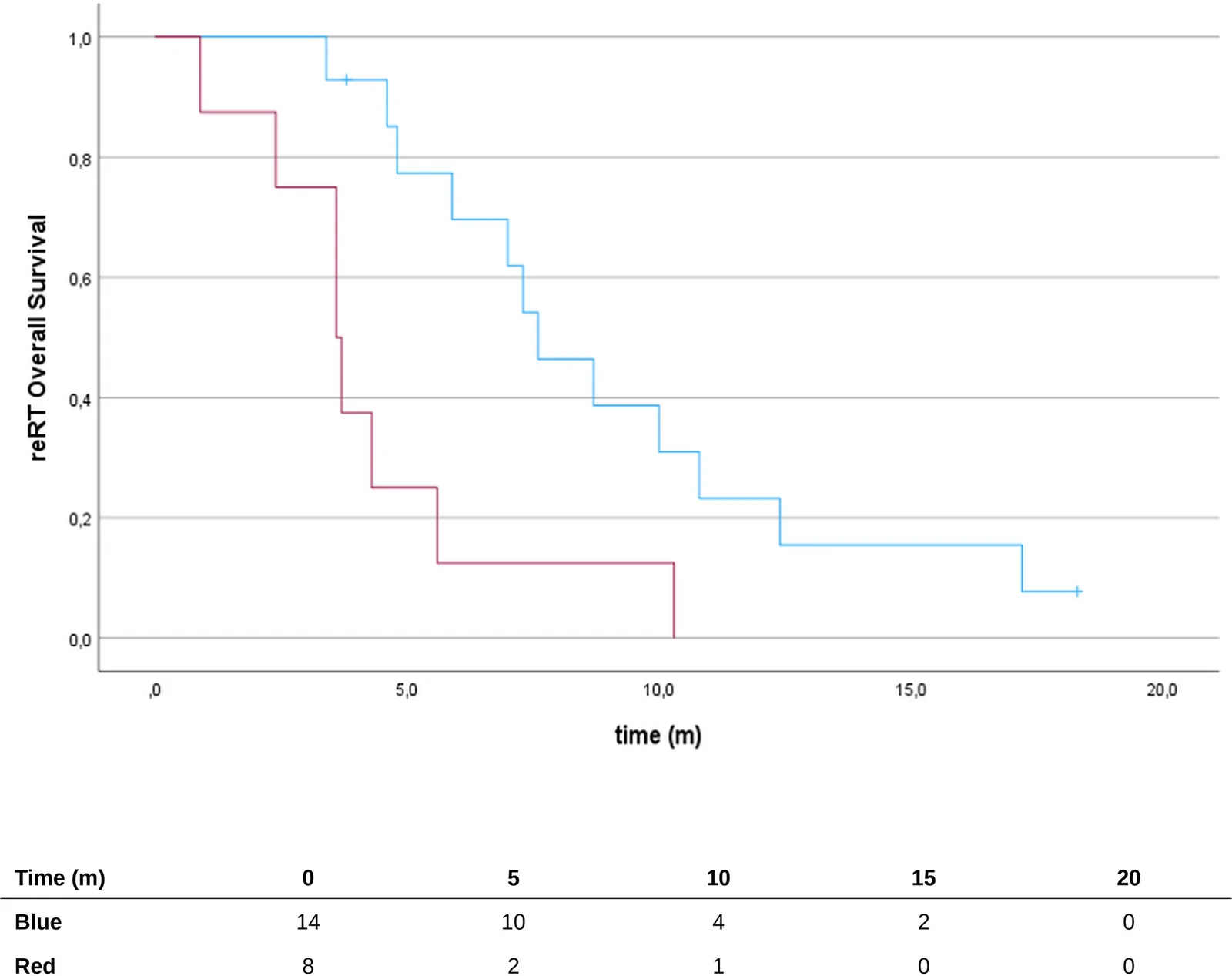
Overall survival RT2 according to radiotherapy doses. Red line, RT2 doses 26–39 Gy; blue line, RT2 doses 50–54 Gy

An association between higher nominal doses and longer survival was observed. The survival benefit of RT2 (*n* = 22) increased with higher nominal doses of radiation (*n* = 14). Also, patients with better clinical status (Lansky/Karnofsky performance scales > or = 50) who received reirradiation doses of 50–54 Gy showed prolonged OS compared to those with lower doses (median 7.3 months vs. 3.6 months; *p* = 0.005) (Fig. 2).

**Fig. 3:**
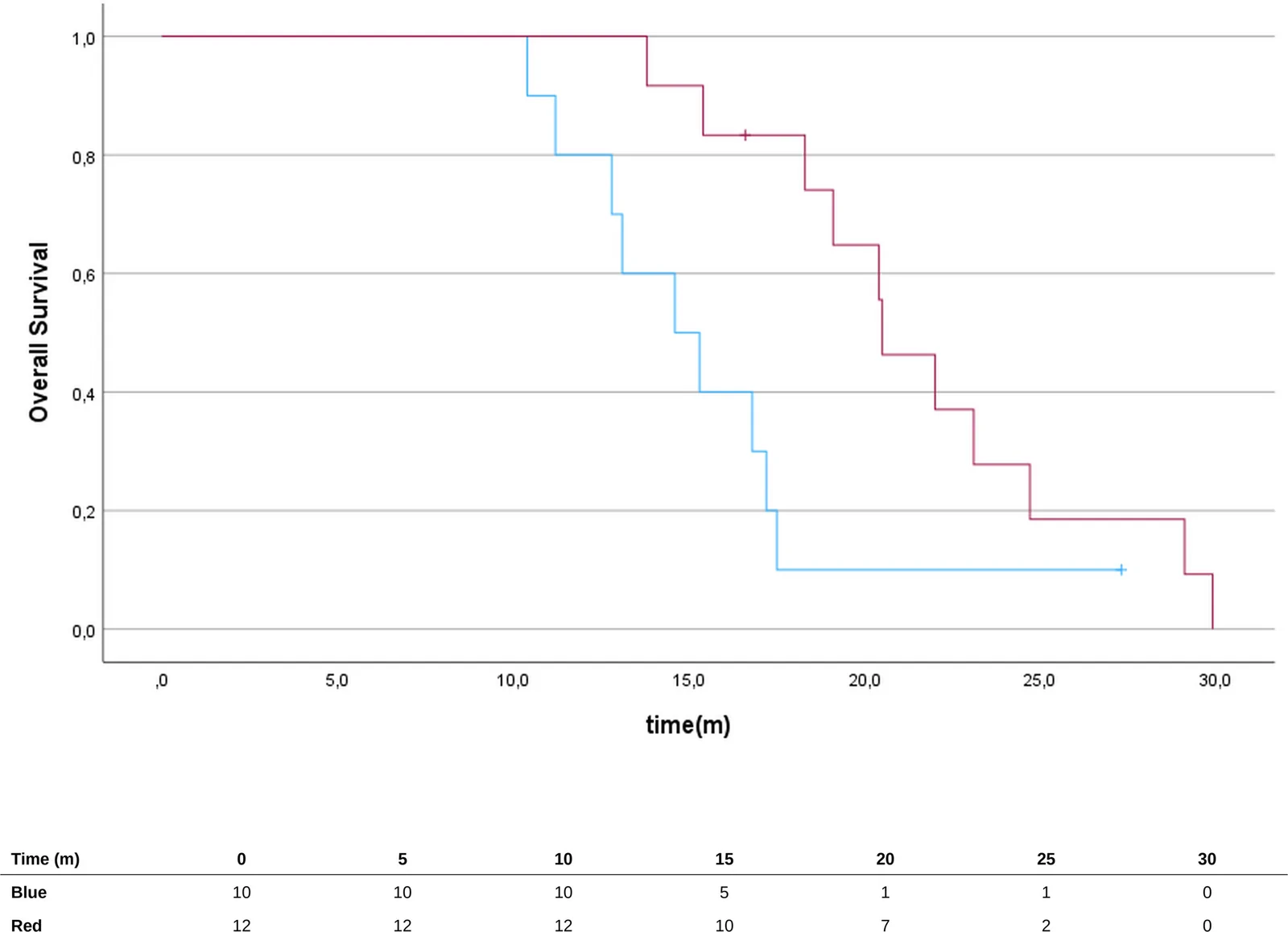
Overall survival according to time after first progression. Red line, patients who progressed after 9 months from the first RT; blue line, patients who progressed earlier than 9 months from the first RT

The survival benefit of reRT (RT2) (*n* = 22) improved with a longer interval from the initial progression (*n* = 12). Patients who experienced progression after 9 months from the first irradiation course had an extended OS following the reirradiation course (RT1 + RT2) compared to those with earlier progression (20.5 months vs. 14.9 months; *p* = 0.019) (Fig. 3).

### Toxicity at reirradiation

Reirradiation was well-tolerated by all patients, with no serious/grade ≥ 3 treatment-related toxicities reported per CTCAE v5.0. Acute toxicities were generally mild (grade 1–2) and included fatigue and transient neurological symptoms*.* Sixteen patients experienced stable and improved neurological function during the treatment. Eight of the 22 patients received bevacizumab to reduce the need for corticosteroids. In two of these eight patients, radionecrosis was detected on MRI, and both showed clinical improvement following bevacizumab treatment.

## Discussion

Our results suggest that full reirradiation for progressive DIPG is well-tolerated when applied to selected patients and with technical rigor using advanced technology. The clinical improvement observed in 75% of patients was comparable to findings in another series of reRTs [14, 15]. Notably, we observed a substantial enhancement in the median OS among patients with a longer time to initial progression and those who received RT2 at higher nominal doses.

An average OS benefit of 3 months has been reported for patients undergoing RT2 compared to those receiving upfront radiotherapy only [12]. The dose–response relation of RT2 is not well established; however, some studies have suggested median OS benefits of 2.7, 3.4, and 4.1 months with doses of 18–20 Gy, 19.8–30 Gy, and 21.6–36 Gy (9). In our study, we demonstrated a median OS benefit of 7.3 months with doses of 50–54 Gy, offering a therapeutic opportunity for these patients in the palliative setting, maintaining the outpatient management approach, and without introducing additional toxicities. These findings must be interpreted with caution given the retrospective, single-center design and the selection bias inherent to the dose assignment process: patients receiving 39 Gy were selected based on poorer performance status and greater clinical fragility, as determined by the treating radiation oncologist. Therefore, more robust patient selection criteria and prospective studies are required to confirm these findings.

Consistent with Janssens et al*.* [16], our cohort showed improved OS among patients with disease progression 6–12 months after initial radiotherapy. Patients experiencing disease progression at 9 months post-initial RT showed enhanced OS following reRT compared to those with shorter progression intervals after the initial RT. Notably, in our cohort, where the minimum interval for reRT was 6 months, outcomes were not significantly influenced by pseudoprogression bias. However, upon analyzing the 11 patients with the shortest time to progression, four received reRT at doses of 39 Gy. This circumstance complicates the determination of whether their shorter OS resulted from the progression interval or the reRT dose. Additionally, these patients presented with the poorest clinical status before RT2 and did not exhibit any clinical improvement post-reRT. Notably, clinical improvement emerged as a crucial prognostic factor for extended OS in the Zaghloul et al. series [5]. This highlights the importance of refining selection criteria for these patients to effectively evaluate the benefits of reRT doses.

Stereotactic biopsies of diffuse brainstem tumors have only been conducted in a limited number of specialized centers. The primary benefit of these biopsies lies in their application in molecular biology. However, the procedure is not devoid of risks. Pincus et al*.* [17] conducted a retrospective study involving 10 stereotactic biopsies — four pontine and six midbrain lesions — and successfully obtained histopathological diagnoses in all cases. One patient experienced a complication leading to diplopia. A review of the literature and an analysis of 13 case series on pediatric brainstem lesions, predominantly located in the pons, revealed diagnostic success rates ranging from 75 to 100%, morbidity rates between 0 and 16%, and mortality rates up to 3.3% [17]. Based on these findings, biopsy is generally recommended for cases exhibiting atypical imaging features or when performed at centers equipped for tumor molecular analysis. Roujeau et al*.* [18] shared their experience with stereotactic biopsy in 24 pediatric patients, reporting no mortality in their series. However, the tissue samples obtained might have been inadequate, potentially leading to inaccurate or missed diagnoses. In our series, we observed no significant adverse events associated with the procedure, with only three out of 26 cases yielding inconclusive IHC analyses due to insufficient material. This demonstrates that, with a proficient pediatric neurosurgery team and adequate resources, biopsy and molecular analyses are feasible, even in resource-constrained settings.

Molecular studies have revealed the heterogeneity of this tumor, with mutations identified in *K27M* (*H3.3* and *H3.1*), *PDGFRA*, *TP53*, *ACVR1*, and *FGFR1* (3,4). Efforts have been undertaken to enhance the understanding of prognostic factors, including the notably poor prognosis linked to the *H3K27* mutation [19], as well as potential therapeutic alternatives. DIPGs frequently exhibit additional genetic mutations, such as *TP53* mutations and *PDGFRA* amplifications, which were also noted in our series. These mutations can impact clinical behavior and prognosis of the tumors. For instance, *TP53* mutations are linked to a higher number of copy variants and poorer survival outcomes [20, 21]. *PDGFRA* amplification has also been recognized as an indicator of unfavorable prognosis in *H3K27M*-mutant tumors [21]. Some DIPGs demonstrate activation of the *MAPK* pathway with mutations in genes such as *FGFR1* and *BRAF* [22]. These changes suggest potential therapeutic targets, although their effect on survival is still under investigation [23].

Despite advances in understanding the molecular mechanisms of DIPG, effective treatments remain elusive. Radiotherapy is the standard of care, but it offers only temporary benefits. Reirradiation is a viable strategy for extending survival. Novel therapeutic approaches, such as targeted therapies and immunotherapies, are currently under investigation [24, 25]. However, access in settings such as low- and middle-income countries (LMIC) is also a significant limitation.

The absence of formal quality-of-life (QoL) assessment instruments represents a meaningful limitation of this study. In the context of a palliative disease such as DIPG, where the primary goal of reirradiation is to extend survival while preserving neurological function and well-being, understanding how survival gains are balanced against treatment burden and QoL is of critical importance. The systematic application of validated QoL scales such as the Pediatric Quality of Life Inventory (PedsQL) was not feasible in our setting, reflecting a broader challenge faced by centers in LMIC, where resource constraints limit the routine implementation of structured QoL tools. Nevertheless, all patients received comprehensive longitudinal follow-up by a multidisciplinary team including pediatric oncologists, radiation oncologists, a dedicated palliative care specialist, psychologists, and allied health professionals. Clinical assessments were conducted at each visit, incorporating neurological evaluation, performance status, symptom burden, and the family’s perspective — providing a meaningful, albeit unstructured, appraisal of functional status and well-being throughout the treatment course.

Furthermore, the assessment of treatment-related toxicity is limited by the retrospective nature of this study, as adverse events were captured through medical record review rather than prospective, systematic evaluation. This approach may result in underreporting subclinical or delayed toxicities, which are less consistently documented in routine clinical follow-up. Prospective studies incorporating formal quality-of-life instruments and structured, longitudinal toxicity assessments at predefined time points are therefore necessary to more rigorously characterize the benefit-risk profile of higher-dose reirradiation in this population. Additionally, this retrospective study encompassed all patients diagnosed with DIPG during the specified period and biopsy has more recently become a standard procedure at our institution, hence the unavailability of these data for all patients. Despite the challenges encountered in LMIC, the findings indicate potential for advancing the comprehension and management of these tumors.

## Conclusion

Our study offers molecular insights into this tumor type and presents a therapeutic option within a palliative context to improve the quality of life and survival of these patients. Given the challenges in accessing targeted therapy in LMIC, reirradiation with higher nominal doses seems to be a safe and viable choice for patients with DIPG post-initial treatment and disease progression. Most patients show neurological improvement and prolonged survival following this approach. The observed survival associations must be interpreted in the context of selection bias and the retrospective study design. Further prospective studies are necessary to obtain enhanced clinical and molecular data to determine which patients would derive benefits from varying reirradiation doses and new targeted drugs.

## Data Availability

No datasets were generated or analysed during the current study.
